# Middle‐ and high‐molecular weight adiponectin levels in relation to nonalcoholic fatty liver disease

**DOI:** 10.1002/jcla.23148

**Published:** 2019-12-27

**Authors:** Kun Lian, Yu‐Nan Feng, Rong Li, Hao‐Lin Liu, Peng Han, Lei Zhou, Cheng‐Xiang Li, Qin Wang

**Affiliations:** ^1^ Department of Cardiology Xijing Hospital The Fourth Military Medical University Xi'an China; ^2^ Department of Geriatrics Xijing Hospital The Fourth Military Medical University Xi'an China; ^3^ Department of Clinical Laboratory Medicine Xijing Hospital The Fourth Military Medical University Xi'an China; ^4^ State Key Laboratory of Cancer Biology Biotechnology Center School of Pharmacy The Fourth Military Medical University Xi'an China

**Keywords:** adiponectin, high‐molecular weight, low‐molecular weight, middle‐molecular weight, nonalcoholic fatty liver disease

## Abstract

**Objective:**

Adiponectin (APN) circulates as high‐molecular weight (HMW), medium‐molecular weight (MMW), and low‐molecular weight (LMW) forms. Nonalcoholic fatty liver disease (NAFLD) is a common cause of chronic liver disease. Currently, the role of LMW, MMW, and HMW APN remains largely unclear in NAFLD.

**Methods:**

We examined the variation of these forms and analyzed the related clinical characteristics in NAFLD. A total of 63 male NAFLD patients (mean age: 43.00 ± 6.10 years) and 70 healthy male subjects (mean age: 42.53 ± 7.98 years) were included in the study. Total APN and other clinical characteristics were measured. The changes in HMW, MMW, and LMW APN were determined in NAFLD patients and NAFLD patients on a high‐fat diet, and the association between the groups was further analyzed.

**Results:**

Decreased levels of total APN and three APN isoforms were found in NAFLD. Significantly decreased levels of HMW (*P* < .01) and MMW (*P* < .001) were observed in NAFLD of high‐fat diet patients. In NAFLD patients, height (*R* = −.270, *P* = .032) and N‐epsilon‐(carboxymethyl) lysine (*R* = −.259, *P* = .040) significantly correlated with total APN. HMW APN was significantly associated with fasting plasma glucose (*R* = .350, *P* = .016), alanine aminotransferase (*R* = −.321, *P* = .029), and aspartate aminotransferase (*R* = −.295, *P* = .045). Additionally, MMW APN was significantly associated with total cholesterol (*R* = .357, *P* = .014) and high‐density lipoprotein (*R* = .556, *P* < .0001). Low‐density lipoprotein (*R* = −.283, *P* = .054) was also clearly associated with LMW APN in NAFLD patients.

**Conclusion:**

These results suggest that HMW and MMW APN may be involved in the pathogenesis and progression of NAFLD.

**Abbreviations**
AGEs: advanced glycation end products
ALT: alanine aminotransferase
AMPK: AMP‐activated protein kinase
APN: adiponectin
AST: aspartate aminotransferase
BCAA: branched‐chain amino acid
BMI: body mass index
CML: N‐epsilon‐(carboxymethyl) lysine
cRAGE: cleaved receptor for advanced glycation end product
DBP: diastolic blood pressure
esRAGE: endogenous secretory receptor for advanced glycation end product
FPG: fasting plasma glucose
HDL‐C: high‐density lipoprotein cholesterol
HMW: high‐molecular weight
LDL‐C: low‐density lipoprotein cholesterol
LMW: low‐molecular weight
MMW: medium‐molecular weight
NAFLD: nonalcoholic fatty liver disease
RA: rheumatoid arthritis
SBP: systolic blood pressure
sRAGE: soluble receptor for advanced glycation end product
T.cho.: total cholesterol
TG: triglycerides

## 1 INTRODUCTION

Nonalcoholic fatty liver disease (NAFLD) is characterized by hepatic fat accumulation of equal to and greater than 5% and is a common cause of chronic liver disease.^1^, ^2^ Nonalcoholic fatty liver disease spans a clinicopathologic spectrum characterized by hepatic steatosis with or without other pathologic features in the absence of other specific causes of fatty liver.^3^, ^4^ Based on the epidemiological data, the global prevalence of NAFLD has been estimated as high as one billion cases.^3^ The development of liver injury in NAFLD is considered a “multiple‐hit process,” which involves many stages, such as triglyceride (TG) and free fatty acid accumulation in hepatocytes, oxidative stress, lipid peroxidation, mitochondrial dysfunction, liver inflammation, insulin resistance, and perturbations of adipokine levels.^5^, ^6^ Extensive studies have reported an association between NAFLD and different indices of insulin resistance, illustrating that either insulin resistance may play a role in the pathogenesis and progression of NAFLD, or that insulin resistance shares a common pathogenic mechanism with liver metabolic disorder.

Adiponectin (APN) is a hormone produced by adipocytes that acts on specific receptors of several tissues through autocrine, paracrine, and endocrine signaling mechanisms. It exists as three distinct and basic oligomeric complexes in plasma as the homotrimer (low‐molecular weight, LMW “mass,” ∼70 kDa) APN, the hexamer (middle‐molecular weight, MMW “mass,” ∼140 kDa) APN, and 12‐18 protomer (high‐molecular weight, HMW “mass,” >300 kDa) APN.^7^, ^8^ Adiponectin plays an important pathophysiological role in metabolic activities such as glucose, lipid, and branched‐chain amino acid metabolism and also functions as an insulin sensitizer. The physiological effects or biological activity of multimer structures of these three forms has recently attracted sufficient attention.^9^, ^10^ Currently, HMW, total APN, and the ratio of HMW APN to total APN have been reported to be associated with diabetes mellitus, insulin resistance, and metabolic syndrome.^10^, ^11^ More importantly, serum APN levels are decreased in NAFLD patients, suggesting that low APN level is an independent risk factor for NAFLD.^12^, ^13^ Significantly lower serum HMW APN level was observed in Taiwanese NAFLD patients with type 2 diabetes.^14^ Different percentages of distribution of the APN isoforms LMW, MMW, and HMW were observed in NAFLD patients.^15^ However, the association of APN isoforms with NAFLD remains largely unclear.

Therefore, the aim of the present study was to investigate the variance in levels of the three oligomers of APN and assess their relation to other parameters in Chinese NAFLD patients.

## 2 MATERIALS AND METHODS

### 2.1 Subjects

The subjects included 63 male NAFLD patients (aged 43.3 ± 6.1 years) and 70 healthy males (aged 42.53 ± 7.98 years) as control subjects undergoing routine health checkups at the Health Examination Center at Xijing Hospital, China. All subjects underwent blood sampling for biochemical studies and an abdominal ultrasonography examination. NAFLD patients met the following inclusion criteria: male; aged 30‐70 years; mild NAFLD; and no medical history of diabetes mellitus, cardiovascular disease, peripheral artery disease, lung, or kidney disease. Subjects were excluded if they had uncontrolled hypertension, serological markers of hepatitis B/C virus infection, autoimmune liver disease, alcoholic liver disease or potential causes of hepatic injury, steatosis, or fibrosis. Age‐matched healthy subjects were included as a control group. The study was approved by the Ethics Committee of Xijing Hospital, The Fourth Military Medical University and each patient provided informed consent.

### 2.2 Blood sampling

Venous blood samples were drawn after fasting; serum samples were separated and analyzed for lipids (total cholesterol [T.cho.], high‐density lipoprotein cholesterol [HDL‐C], low‐density lipoprotein cholesterol [LDL‐C], and TG), fasting plasma glucose (FPG), aspartate aminotransferase (AST), alanine aminotransferase (ALT), soluble receptor for advanced glycation end product (sRAGE), branched‐chain amino acid (BCAA), and N‐epsilon‐(carboxymethyl) lysine (CML) within 48 hours. For the APN assay, aliquots of samples were stored at −80°C. Total APN, sRAGE, CML, and BCAA were determined using a commercially available enzyme‐linked immunosorbent assay (ELISA) kit according to the manufacturer's instructions (R&D systems). The intra‐assay and inter‐assay coefficients of variation were <6 and <8%, respectively.

### 2.3 Analysis of adiponectin multimers

Adiponectin multimers were analyzed by Western blotting followed by sodium dodecyl sulfate (SDS)‐polyacrylamide gel electrophoresis. Adiponectin in serum was diluted 10 times and combined with 5× Laemmli sample buffer without a reducing agent. Samples (15 μL) were loaded and run in 4%‐15% criterion precast 10‐well gels (Bio‐Rad). Following electrophoresis, representative samples were transferred to nitrocellulose membranes (PALL) and subjected to immunoblotting analysis. Monoclonal anti‐human/mouse Acrp30/APN (R&D Systems) and goat anti‐rat IgG horseradish peroxidase (Santa Cruz Biotechnology) antibodies were used for Western blot analyses. The blots were developed using an ECL‐Plus chemiluminescence reagent kit (Amersham Bioscience) and visualized using a UVP Bio‐Imaging System. Blot densities were analyzed using Vision Works LS Acquisition and Analysis Software.

### 2.4 Statistical analysis

Statistical analysis of the data was performed using SPSS Statistics V22.0 (SPSS Inc). The continuous variables were presented as the mean ± SD. The comparisons of the means between the two groups were tested by a Student's *t* test, and correlations among the parameters were tested by Pearson's correlation coefficient. A *P* value of <.05 was considered as statistically significant.

## 3 RESULTS

As shown in Table 1, the general clinical characteristics, including weight, body mass index (BMI), systolic blood pressure (SBP), diastole blood pressure (DBP), total APN, BCAA, CML, FPG, T.cho., TG (all *P* < .001), LDL‐C (*P* < .01), and HDL‐C (*P* < .05), were significantly higher in NAFLD patients when compared with age‐matched healthy control subjects. Contrastingly, the levels of sRAGE (*P* < .05) and APN (*P* < .01) were significantly decreased in NAFLD patients. The two groups did not differ in the parameters of age, height, AST, or ALT.

**Table 1:** General characteristics of the study population

Characteristics	Control subjects (n = 70)	NAFLD patients (n = 63)	*P* value
Age (y)	42.53 ± 7.98	43.00 ± 6.10	.7057
Height (m)	1.73 ± 0.06	1.74 ± 0.06	.3390
Weight (Kg)	68.79 ± 8.29	79.08 ± 8.06	<.0001
BMI (kg/m^2^)	23.00 ± 2.28	26.19 ± 2.29	<.0001
SBP (mm Hg)	110.40 ± 12.23	117.06 ± 12.63	.0024
DBP (mm Hg)	72.10 ± 9.38	76.78 ± 9.39	.0048
AST (IU/L)	21.47 ± 7.73	22.28 ± 5.68	.4963
ALT (IU/L)	22.30 ± 10.14	24.43 ± 9.37	.2122
Total APN (ug/mL)	5.85 ± 3.74	4.26 ± 2.71	.0062
Plasma BCAA	524.07 ± 101.78	672.51 ± 86.30	<.0001
CML (pg/mL)	63.28 ± 24.78	78.42 ± 30.53	.0020
sRAGE (pg/mL)	996.41 ± 440.85	844.04 ± 348.14	.0299
FPG (mmol/mL)	4.70 ± 0.39	5.01 ± 0.54	.0002
T.cho. (mmol/L)	4.10 ± 0.67	4.71 ± 0.78	<.0001
TG (mmol/mL)	1.15 ± 0.41	1.62 ± 0.67	<.0001
LDL (mmol/mL)	1.59 ± 0.49	1.83 ± 0.51	.0065
HDL (mmol/mL)	1.30 ± 0.23	1.39 ± 0.28	.0441

Note

Values are represented as mean ± SD.

Abbreviations: ALT, alanine aminotransferase; APN, adiponectin; AST, aspartate aminotransferase; BCAA, branched‐chain amino acid; BMI, body mass index; CML, N‐epsilon‐(carboxymethyl) lysine; DBP, diastolic blood pressure; FPG, fasting plasma glucose; HDL‐C, high‐density lipoprotein cholesterol; LDL, low‐density lipoprotein cholesterol; NAFLD, nonalcoholic fatty liver disease; SBP, systolic blood pressure; sRAGE, soluble receptor for advanced glycation end product; T.cho., total cholesterol; TG, triglycerides.

**Figure 1:**
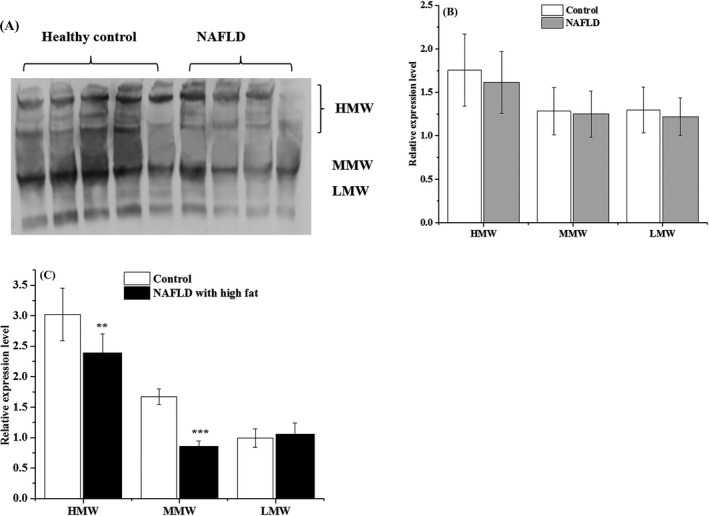
Relative expression levels of LMW, MMW, and HMW APN isoforms. A, Representative photograph of different APN isoform expression via Western blot analysis in control and NAFLD groups. B, The corresponding statistical analysis of relative expression of different APN isoforms (control n = 34, NAFLD n = 47). C, The relative expression of different APN isoforms of NAFLD patients on high‐fat diets and the corresponding control subjects (control n = 6, NAFLD on high‐fat diet n = 6). **P < .01, ***P < .001 when compared with the corresponding control group

In this study, 34 control subjects and 47 NAFLD patients were further analyzed for APN isoforms. A representative photograph of different APN isoform expression is shown in Figure 1A. We found that serum HMW, MMW, and LMW APN levels were lower in NAFLD patients (Figure 1B). Subsequently, NAFLD patients following high‐fat diets were separated from NAFLD patients according to the fat intake level in their daily diet. We further determined the levels of APN isoforms in NAFLD patients on high‐fat diets and the control subjects, and found that serum HMW and MMW APN were significantly lower (*P* < .01 and *P* < .001, respectively) in NAFLD patients on a high‐fat diet compared with those in the corresponding control subjects (Figure 1C).

In the simple correlation analysis of the whole data set, serum total APN and the APN isoforms of HMW, MMW, and LMW were analyzed according to other clinical characteristics indicated in Tables 2, 3, 4, 5. Total APN levels were positively associated with age (*R* = .243, *P* = .042), negatively associated with TG (*R* = −.250, *P* = .037), LDL‐C (*R* = −.264, *P* = .027) in control subjects, and negatively associated with height (*R* = −.270, *P* = .032) and CML (*R* = −.259, *P* = .040) in NAFLD patients (Table 2). Furthermore, we analyzed the correlation between the APN isoforms of HMW, MMW, LMW, and other clinical characteristics to identify which factors affect the concentrations of these isoforms in control and NAFLD patients. As shown in Table 3, we found that BMI and DBP were positively associated with HMW APN in control subjects (*R* = .430, *P* = .011; *R* = .370, *P* = .031, respectively), similar to FPG (*R* = .350, *P* = .016) in NAFLD patients, while HMW APN was significantly and negatively correlated with ALT (*R* = −.321, *P* = .029) and AST (*R* = −0.295, *P* = .045) in NAFLD patients. As Table 4 demonstrates, positive correlations with MMW APN were observed in T.cho. (*R* = .357, *P* = .014) and HDL‐C(*R* = .556, *P* < .0001) in NAFLD patients. As indicated in Table 5, LDL‐C was in obvious and inverse association (*R* = −0.283, *P* = .054) with LMW APN in NAFLD patients.

**Table 2:** Correlation of the factors associated with plasma total APN levels

Characteristics^a^	Control subjects (n = 70) *R* (*P* value)	NAFLD patients (n = 63) *R* (*P* value)
Age (y)	.243 (.042)	−.045 (.724)
Height (m)	−.102 (.401)	−.270 (.032)
Weight (kg)	−.107 (.379)	−.127 (.323)
BMI (kg/m^2^)	−.055 (.654)	−.143 (.262)
SBP (mm Hg)	.231 (.054)	.064 (.618)
DBP (mm Hg)	.175 (.148)	.131 (.308)
AST (IU/L)	−.114 (.346)	−.059 (.645)
ALT (IU/L)	.035 (.773)	−.094 (.465)
BCAA	.035 (.829)	−.170 (.195)
CML (pg/mL)	−.175 (.148)	−.259 (.040)
sRAGE (pg/mL)	.104 (.390)	.022 (.862)
FPG (mmol/mL)	−.006 (.963)	−.082 (.520)
T.cho. (mmol/mL)	.079 (.517)	.133 (.300)
TG (mmol/mL)	−.250 (.037)	−.064 (.618)
LDL‐C (mmol/mL)	−.264 (.027)	.036 (.778)
HDL‐C (mmol/mL)	.046 (.707)	.114 (.375)

Note

Values are represented as mean ± SD.

Abbreviations: ALT, alanine aminotransferase; AST, aspartate aminotransferase; BCAA, branched‐chain amino acid; BMI, body mass index; CML, N‐epsilon‐(carboxymethyl) lysine; DBP, diastolic blood pressure; FPG, fasting plasma glucose; HDL‐C, high‐density lipoprotein cholesterol; LDL, low‐density lipoprotein cholesterol; NAFLD, nonalcoholic fatty liver disease; SBP, systolic blood pressure; sRAGE, soluble receptor for advanced glycation end product; T.cho., total cholesterol; TG, triglycerides.

a Data were compared with the BCAAs via Pearson's correlation test.

**Table 3:** Correlation of the factors associated with plasma HMW APN levels

Characteristics^a^	Control subjects (n = 34) *R* (*P* value)	NAFLD patients (n = 47) *R* (*P* value)
Age (y)	.020 (.912)	.122 (.412)
Height (m)	−.027 (.878)	−.137 (.358)
Weight (kg)	.311 (.074)	−.002 (.990)
BMI (kg/m^2^)	.430 (.011)	.114 (.446)
SBP (mm Hg)	.244 (.165)	.187 (.208)
DBP (mm Hg)	.370 (.031)	.179 (.228)
AST (IU/L)	−.188 (.287)	−.295 (.045)
ALT (IU/L)	.136 (.444)	−.321 (.029)
BCAA	.167 (.345)	−.223 (.132)
CML (pg/mL)	.176 (.320)	.015 (.923)
sRAGE (pg/mL)	.103 (.563)	−.033 (.827)
FPG (mmol/mL)	−.068 (.703)	.350 (.016)
T.cho. (mmol/mL)	−.231 (.188)	.050 (.739)
TG (mmol/mL)	−.028 (.875)	−.179 (.229)
LDL‐C (mmol/mL)	−184 (.298)	−.0033 (.823)
HDL‐C (mmol/mL)	−.161 (.363)	.000 (.998)

Note

Values are represented as mean ± SD.

Abbreviations: ALT, alanine aminotransferase; AST, aspartate aminotransferase; BCAA, branched‐chain amino acid; BMI, body mass index; CML, N‐epsilon‐(carboxymethyl) lysine; DBP, diastolic blood pressure; FPG, fasting plasma glucose; HDL‐C, high‐density lipoprotein cholesterol; LDL, low‐density lipoprotein cholesterol; NAFLD, nonalcoholic fatty liver disease; SBP, systolic blood pressure; sRAGE, soluble receptor for advanced glycation end product; T.cho., total cholesterol; TG, triglycerides.

a Data were compared with the BCAAs via Pearson's correlation.

**Table 4:** Correlation of the factors associated with plasma MMW APN levels

Characteristics^a^	Control subjects (n = 34) *R* (*P* value)	NAFLD patients (n = 47) *R* (*P* value)
Age (y)	−.312 (.072)	−.004 (.981)
Height (m)	−.019 (.913)	.131 (.180)
Weight (kg)	−.001 (.998)	.020 (.892)
BMI (kg/m^2^)	.011 (.950)	−.046 (.757)
SBP (mm Hg)	−.043 (.809)	.062 (.678)
DBP (mm Hg)	.107 (.547)	.045 (.766)
AST (IU/L)	−.031 (.862)	.042 (.777)
ALT (IU/L)	−.157 (.377)	−.158 (.290)
BCAA	−.118 (.506)	−.020 (.893)
CML (pg/mL)	−.011 (.953)	.247 (.095)
sRAGE (pg/mL)	.009 (.959)	−.073 (.628)
FPG (mmol/mL)	−.024 (.893)	−.159 (.286)
T.cho. (mmol/mL)	−.027 (.880)	.357 (.014)
TG (mmol/mL)	.111 (.532)	−.101 (.497)
LDL‐C (mmol/mL)	−.065 (.714)	.207 (.163)
HDL‐C (mmol/mL)	−.120 (.499)	.556 (.0001)

Note

Values are represented as mean ± SD.

Abbreviations: ALT, alanine aminotransferase; AST, aspartate aminotransferase; BCAA, branched‐chain amino acid; BMI, body mass index; CML, N‐epsilon‐(carboxymethyl) lysine; DBP, diastolic blood pressure; FPG, fasting plasma glucose; HDL‐C, high‐density lipoprotein cholesterol; LDL, low‐density lipoprotein cholesterol; NAFLD, nonalcoholic fatty liver disease; SBP, systolic blood pressure; sRAGE, soluble receptor for advanced glycation end product; T.cho., total cholesterol; TG, triglycerides.

a Data were compared with the BCAAs via Pearson's correlation.

**Table 5:** Correlation of the factors associated with plasma LMW APN levels

Characteristics^a^	Control subjects (n = 34) *R* (*P* value)	NAFLD patients (n = 47) *R* (*P* value)
BMI (kg/m^2^)	.108 (.542)	.091 (.542)
SBP (mm Hg)	.160 (.365)	.117 (.433)
DBP (mm Hg)	.229 (.192)	.103 (.490)
AST (IU/L)	.172 (.329)	−.162 (.277)
ALT (IU/L)	.005 (.977)	.069 (.644)
BCAA	.108 (.543)	.045 (.762)
CML (pg/mL)	−.046 (.786)	.034 (.819)
sRAGE (pg/mL)	.294 (.091)	.049 (.743)
FPG (mmol/mL)	−.020 (.913)	.025 (.865)
T.cho. (mmol/mL)	−.002 (.991)	−.177 (.235)
TG (mmol/mL)	.022 (.900)	−.257 (.082)
LDL‐C (mmol/mL)	−.117 (.511)	−.283 (.054)
HDL‐C (mmol/mL)	.181 (.306)	−.064 (.669)

Note

Values are represented as mean ± SD.

Abbreviations: ALT, alanine aminotransferase; AST, aspartate aminotransferase; BCAA, branched‐chain amino acid; BMI, body mass index; CML, N‐epsilon‐(carboxymethyl) lysine; DBP, diastolic blood pressure; FPG, fasting plasma glucose; HDL‐C, high‐density lipoprotein cholesterol; LDL, low‐density lipoprotein cholesterol; NAFLD, nonalcoholic fatty liver disease; SBP, systolic blood pressure; sRAGE, soluble receptor for advanced glycation end product; T.cho., total cholesterol; TG, triglycerides.

a Data were compared with the BCAAs via Pearson's correlation.

## 4 DISCUSSION

Metabolic syndrome is a cluster of metabolic abnormalities including diabetes, cardiovascular disease, and NAFLD.^16^ In this study, we have reached the following findings: First, we found that the total APN and the distribution of APN isoforms were decreased in NAFLD patients. Second, in NAFLD patients following a high‐fat diet, significantly decreased levels of HMW and MMW were observed, while the plasma BCAA levels were significantly increased. Third, CML was significantly negatively correlated with total APN in NAFLD patients, which is in alignment with a study conducted by Del Turco, et al^17^ The corresponding sRAGE levels indicated a significant decrease in NAFLD patients, which was in positive association with total APN. Fourth, HMW APN was significantly positively associated with FPG but negatively correlated with ALT and AST. Last, MMW APN was significantly positively associated with T.cho and HDL, while LMW APN was negatively correlated with LDL. These results suggest that total, HMW, MMW, and LMW APN levels may descend with the pathological progression in NAFLD patients.

Adiponectin is involved in the development of insulin resistance. It has been confirmed that plasma APN levels are positively associated with insulin resistance and type 2 diabetes. However, many research studies have confirmed that metabolic syndrome is in negative association with APN. HMW APN, which functions to activate the AMP‐activated protein kinase (AMPK) signaling pathway in the liver as target organ, is the predominant form of endogenous APN related to metabolic effects.^18^ Additionally, Bianchi et al^15^ concluded that APN levels were reduced in NAFLD patients, without any significant contribution of isoform distribution to progressive liver disease. We found that total APN and three APN isoforms were inversely correlated with NAFLD in this study, which is an additional supplement to their study. The association among APN isotypes may be attributed to the following reasons: First, the subjects included in this study were from the Chinese population, while those in the former study were from European populations. Second, the measurement of APN concentration was different; we used the semi‐quantitative method, Western blot analysis, while they used an in‐house validated time‐resolved immunofluorometric assay and a fast protein liquid chromatography (FPLC) assay.^15^ However, a larger sample size is warranted to prove the association among different APN isoforms demonstrated in our study.

Branched‐chain amino acids are a collection of essential amino acids such as leucine, isoleucine, and valine. Their homeostasis is greatly affected by catabolic organs and tissues, such as liver, muscle, and adipose tissue. Metabolic syndrome has a strong correlation with selective BCAA profile disturbances, whose availability affects glucose, protein, and lipid metabolism.^19^ Zhang, et al^20^ demonstrated that BCAAs triggered abnormal lipolysis and hyperlipidemia, causing hepatic lipotoxicity. Furthermore, BCAAs directly exacerbate hepatic lipotoxicity by reducing lipogenesis and inhibiting autophagy in the hepatocyte. Here, we discovered that BCAAs are significantly higher in NAFLD patients compared with age‐matched healthy control subjects. This result testified that the increasing levels of circulating BCAAs may be the injury factor to the liver. In our previous study, we found that APN deficiency contributed to impaired BCAA catabolism by decreasing branched‐chain alpha‐keto acid dehydrogenase activity via the AMPK‐PP2Cm signaling pathway.^21^ Thus, decreased APN may be the cause of elevated BCAA levels. In this study, we observed significantly elevated serum BCAA levels and significantly reduced APN levels in the NAFLD patients, but the association between circulating BCAA and APN in the Chinese population was not significant. This result could be attributed to the specific population, the small sample size, and the specific detection method. The result should be confirmed by a more comprehensive planned investigation to prove the consistent correlation between BCAAs and APN.

N‐epsilon‐(carboxymethyl) lysine is a major component of advanced glycation end products (AGEs), which is a heterogeneous group of molecules formed by the nonenzymatic reaction of reducing sugars with the amino group of proteins, lipids, and nucleic acids.^22^, ^23^ Soluble receptor for AGE (sRAGE) binds with AGEs as a competing factor for RAGE, and sRAGE consists of both cleaved RAGE (cRAGE) and endogenous secretory RAGE (esRAGE) which circulate in the blood.^24^ The formation of AGEs is an important biochemical abnormality along with many pathological conditions involving inflammation, hyperglycemia, and oxidative stress. Ivancovsky‐Wajcman et al^25^ have suggested that serum sRAGE levels are associated with NAFLD that may function as a protective factor against RAGE in the AGEs‐RAGE/sRAGE system. Moreover, previous studies showed that CML accumulates much more pronounced in human fatty livers,^26^, ^27^, ^28^ where it is decomposed and secreted into the bloodstream as an unstable component. However, the association of circulating CML in serum with NAFLD is not known. In our study, we discovered that the CML level was decreased and the sRAGE level elevated in the serum of NAFLD patients. Thus, our research further confirms that the AGEs‐RAGE/sRAGE system plays an important role in the pathogenesis of NAFLD and the progression of liver injury.^29^

Adiponectin exerts a significant effect on glucose and lipid metabolism, where the different isoforms which possess distinct biological properties, activate specific signaling pathways.^13^ Similar to previous descriptions,^12^, ^13^ a significant reduction of total APN levels was found in NAFLD patients in our study. Simultaneously, significantly increased or an elevating trend of BMI, CML, FPG, T.cho., TG, and LDL‐C levels was found, as summarized by Neuman.^12^ Although HDL‐C was previously found to be lower in NAFLD patients,^12^ the mean ratio of TG/HDL‐C was increased from control (0.885) to NAFLD (1.165) patients. Higher lipid ratios indicated a significantly greater risk for advanced NAFLD patients.^30^ Furthermore, patients with a higher LDL‐C level within the normal range had an increased cumulative incidence rate of NAFLD.^31^

In our study, total APN and its different isoforms demonstrated correlations with the different relevant factors of NAFLD. As with NAFLD, decreased HMW APN was also reported in asthma, rheumatoid arthritis (RA), type 2 diabetes, and NAFLD patients.^11^, ^15^, ^32^, ^33^ Although reports have demonstrated that an inverse relationship between plasma APN, which suppresses hepatic glucose output, and endogenous glucose production exists in NAFLD patients,^36^ we found that HMW APN was significantly positively associated with FPG in our study. The diversion of correlation may be attributed to the differences between the distinctive populations and the small number of subjects in our study. In contrast, ALT and AST, the two aminotransferases were significantly reversely correlated with HMW APN in NAFLD patients. Similar to our study, previous studies have demonstrated that even within the normal range, an independent inverse association exists between ALT and HMW APN in diabetics, childhood obesity, and NAFLD patients with BMI <25 kg/m^2^.^34^, ^35^, ^36^ Our finding provided additional evidence that AST is significantly and inversely correlated with HMW APN in NAFLD patients with no diabetes mellitus and obesity. Thus, the variation of HMW APN, rather than the MMW and LMW APN isoforms, is more correlated with liver function.

In previous investigations, MMW and LMW APN have not been studied sufficiently. In our study, both the MMW and LMW APN isoforms declined in NAFLD patients. T.cho. and HDL were more significantly positively associated with MMW APN than with other clinical characteristics. Previously, decreased LMW APN was associated with serum TG in episodes of asthma.^32^ Additionally, lower levels of LMW APN were found in RA, type 2 diabetes, and NAFLD patients.^9^, ^11^, ^15^ In our research, it is also indicated that LMW APN was clearly negatively affected by LDL in NAFLD patients that is consistent with the significant reduction of APN in NAFLD patients on high‐fat diets. Currently, HMW APN is suggested to be more important in lipid metabolism in diabetics or metabolically unhealthy subjects,^37^, ^38^ while our findings may suggest the important role of MMW APN in lipid metabolism in NAFLD patients, especially those on high‐fat diets.

There are several limitations to our study. First, we used Western blot analysis under non‐heating and non‐reducing conditions for the detection of APN distributions to determine relative APN isoform levels. Second, the sample size in our investigation was relatively small and the studied participants were limited to Chinese individuals from adjacent districts and males only, since our research group had concluded that significant differences of plasma BCAAs existed in different genders in another research project. Additionally, the number of NAFLD patients on high‐fat diets was smaller than that in other groups. Further studies are needed to confirm these important results in larger clinical studies. Third, this is a cross‐sectional study based on the observation of clinical characteristics, where merely the correlation between APN isoforms and NAFLD was possible to be observed rather than the causal associations. Thus, longitudinal studies should be adopted in further investigations as an approach to identify causal associations.

In conclusion, our findings suggest that decreased total, HMW, MMW, and LMW APN levels were observed in NAFLD patients. Height and CML were significantly correlated with total APN. These results suggest that HMW and MMW APN may play an important role in the pathogenesis and progression of NAFLD. In addition, HMW APN and MMW APN may be closely associated with liver function and lipid metabolism, respectively, and can be considered potential novel therapeutic approaches for NAFLD.
